# Application of Engineered Nanomaterials as Nanocatalysts in Catalytic Ozonation: A Review

**DOI:** 10.3390/ma17133185

**Published:** 2024-06-28

**Authors:** Rita M. F. Cardoso, Joaquim C. G. Esteves da Silva, Luís Pinto da Silva

**Affiliations:** 1 Chemistry Research Unit (CIQUP), Institute of Molecular Sciences (IMS), Department of Geosciences, Environment and Spatial Plannings, Faculty of Sciences, University of Porto, Rua do Campo Alegre s/n, 4169-007 Porto, Portugal; up201704723@edu.fc.up.pt (R.M.F.C.); jcsilva@fc.up.pt (J.C.G.E.d.S.); 2 LACOMEPHI, GreenUPorto, Department of Geosciences, Environment and Spatial Plannings, Faculty of Sciences, University of Porto, Rua do Campo Alegre s/n, 4169-007 Porto, Portugal

**Keywords:** engineered nanomaterials, wastewater treatment, advanced oxidation processes, catalytic ozonation, nanocatalysts

## Abstract

Given the growing scarcity of water and the continuous increase in emerging pollutants detected in water bodies, there is an imperative need to develop new, more effective, and sustainable treatments for wastewater. Advanced oxidation processes (AOPs) are considered a competitive technology for water treatment. Specifically, ozonation has received notable attention as a promising approach for degrading organic pollutants in wastewater. However, different groups of pollutants are hardly degradable via single ozonation. With continuous development, it has been shown that using engineered nanomaterials as nanocatalysts in catalytic ozonation can increase efficiency by turning this process into a low-selective AOP for pollutant degradation. Nanocatalysts promote ozone decomposition and form active free radicals responsible for increasing the degradation and mineralization of pollutants. This work reviews the performances of different nanomaterials as homogeneous and heterogeneous nanocatalysts in catalytic ozonation. This review focuses on applying metal- and carbon-based engineered nanomaterials as nanocatalysts in catalytic ozonation and on identifying the main future directions for using this type of AOP toward wastewater treatment.

## 1. Introduction

Water is a fundamental resource for human life, and the current imbalance between demand for water and its availability is becoming increasingly worrying. This imbalance has arisen due to growing industrialization, an increase in the global population, continuous economic development, and climate change, leading to an increase in the concentration of numerous pollutants in water [1,2]. The unsustainable exploitation of water resources results not only in water scarcity, but also in a deterioration in its quality, a serious environmental problem facing the world today [3,4].

In recent decades, there has been a worrying increase in the concentrations of organic and inorganic compounds in the aquatic environment, posing significant risks to human health and the integrity of ecosystems. Pesticides, pharmaceuticals, and dyes are among the compounds that stand out most negatively in this regard, as they are widely used in industry, are difficult substances to degrade, and consequently have a high prevalence in wastewater [5]. The lack of worldwide regulation of these emerging contaminants further aggravates the situation, highlighting the urgency of implementing preventive and corrective actions in water quality management and developing sustainable processes and more effective water treatment technologies to tackle this environmental problem [2,5].

Water contaminated by organic compounds is often treated using conventional treatment methods, including coagulation, flocculation, adsorption, reverse osmosis, membrane separation, electrocoagulation, ion exchange, desalination, and biological processes [6]. However, most of these methods have limitations in their applicability and efficiency, failing to remove or reduce the concentrations of toxic and/or recalcitrant organic substances from wastewater to below-acceptable levels. There is, therefore, a substantial need to develop new, alternative, and more effective wastewater treatment technologies [7,8].

Advanced oxidation processes (AOPs) have been gaining prominence in water treatment and are currently recognized as an emerging and promising technology for degrading persistent organic pollutants that are refractory to other environmental remediation treatments. AOPs are based on producing reactive oxygen species (ROS) to decompose and mineralize numerous aqueous contaminants, converting them into relatively benign substances [7,9].

AOPs involve the formation of highly reactive free radicals, such as hydroxyl radical (OH^•^) and sulfate radical (SO4^•−^). These radicals are the main oxidants responsible for the enhanced performance of AOPs compared to conventional treatment methods. They are extremely reactive and non-selective oxidants which are capable of reacting with numerous classes of organic compounds in water. Oxidants are known for their high reaction rate, which provides an effective and environmentally conscious solution for wastewater treatment [10,11].

Given their significant performance, AOPs are currently considered competitive techniques in wastewater management and are, therefore, used in water treatment for the removal of specific pollutants, sludge treatment, and color and odor reduction [8]. AOPs promote increased biodegradability and detoxification of wastewater, transforming organic contaminants into less toxic and easily degradable or even non-toxic products such as CO_2_ and H_2_O. In this way, AOPs can provide a definitive solution for wastewater treatment. However, it is important to consider that, in some cases, the oxidation products can be more toxic than the original contamination, which requires careful evaluation of the by-products formed during treatment. AOPs are recognized as versatile and environmentally friendly technologies, providing alternatives for generating reactive oxygen species [8,9,12,13].

Various advanced homogeneous and heterogeneous AOPs have been developed and applied in wastewater treatment in recent years. The most widely investigated AOPs include the Fenton system (Fe^2+^/H_2_O_2_), photo-Fenton system (Fe^2+^/H_2_O_2_/UV), heterogeneous photocatalytic oxidation, ozone (O_3_)-based processes, electrochemical oxidation, and ultrasound [8]. Among the various AOPs, ozonation is an imperative technology for oxidizing and removing various refractory substances in wastewater. Since the end of the 19th century, ozonation has been widely applied in wastewater remediation, particularly for the treatment of textile effluents [8,14].

O_3_ is a powerful oxidizer with numerous benefits for use in AOPs. When dissolved in water, O_3_ acts as an oxidizer due to its high oxidation potential (E^θ^ = 2.07 V), effectively breaking down a wide range of organic and inorganic compounds. In addition to its high efficiency in degradation, ozonation is also useful in removing color and controlling taste and odor problems in effluents [15]. Ozone-based processes are considered environmentally friendly and versatile and can be easily implemented in pre-existing wastewater treatment plants [15,16].

In water, O_3_ undergoes a series of reactions, decomposing into various oxidative species through different pathways, namely, through molecular O_3_ (direct pathway), which reacts with organic contaminants, or through the hydroxyl radical (indirect pathway), which is responsible for oxidizing organic pollutants [15,17]. These reactions depend on various variables, such as the nature of the compound, the dose of O_3_, and the pH of the medium. As a rule, direct ozonation predominates under acidic conditions, while indirect ozonation prevails under alkaline conditions, the latter being the most important as it is the main route responsible for destroying numerous refractory compounds [8,18,19].

However, O_3_ as an oxidant can show some selectivity, and different groups of pollutants are hardly degradable via single ozonation. Thus, the efficiency of ozonation needs to be improved, and its selectivity reduced. The solution to this problem can be coupling engineered nanomaterials with O_3_ in so-called catalytic ozonation, in which the former act as nanocatalysts. The addition of engineered nanomaterials can catalyze the decomposition of O_3_ and the formation of the highly reactive hydroxyl radical [20]. This makes this process less selective toward the degradation of organic pollutants while increasing degradation yields and reducing environmental impacts. In recent years, engineered nanomaterials have been shown to be promising nanocatalysts for ozonation, resulting in a relevant increase in the efficiency of this AOP and elimination of the chemical substances involved in these treatments.

Nanomaterials are materials with at least one nanometric size dimension (0.1–100 nm) [11,20,21]. The unique properties of nanomaterials give them a high adsorption capacity, making them highly effective in accelerating oxidation reactions during the ozonation process [3,22]. Nanotechnology thus makes it possible to reduce or eliminate the number of chemical substances involved in the ozonation process, significantly reducing the environmental impacts associated with these processes [21,22].

Nanomaterials are emerging as a promising approach to water treatment, offering a wide range of applications and thus playing a crucial role in environmental remediation. Several nanoparticles have been successfully developed to degrade different organic pollutants present in wastewater. One study synthesized copper sulfide (CuS)-based nanoparticles that showed high efficacy in degrading dyes [23]. A highly active and magnetically re-extractable palladium nanocatalyst (Pd/Fe_3_O_4_) was developed, showing great potential for application in wastewater treatment processes [24]. To date, several nanomaterials have been successfully applied in catalytic ozonation. Metal/metal oxide-based nanomaterials and carbon-based nanomaterials have aroused considerable interest from the scientific community due to their exceptional characteristics, which make them ideal catalysts to be coupled with ozonation in wastewater treatment [22,25,26].

Water treatment technologies must be constantly improved to meet increasingly stringent water quality standards [11]. Incorporating nanocatalysts into more complex treatment technologies can increase the removal efficiency of a wide range of pollutants, achieving the desired water quality objectives. One study showed that sonication-assisted catalytic ozonation significantly improves the removal of microplastics from water, demonstrating the effectiveness of this integrated approach in wastewater treatment [27].

The aim of this article is to review the performances of different nanomaterials that have been applied as homogeneous and heterogeneous nanocatalysts in catalytic ozonation. More specifically, we present and analyze the main metal/metal oxide-based nanocatalysts and carbon-based nanocatalysts currently being proposed to be coupled with catalytic ozonation for the removal/degradation of organic compounds in wastewater.

## 2. Metal/Metal Oxide-Based Catalysts

Numerous metal-based catalysts are widely applied in the heterogeneous catalytic ozonation process for wastewater treatment. Metal oxides such as MgO, CuO, TiO_2_, ZnO, Al_2_O_3_, NiO, MnO_2_, and FeOOH are among the most studied catalysts for the degradation of organic pollutants in aqueous solutions due to their high surface areas and excellent catalytic properties, which provide faster and more effective adsorption and degradation of pollutants [16,26,28,29]. Some examples of metal/metal oxide-based nanocatalysts used in the ozonation process are summarized in Table 1.

### 2.1. Iron-Based Catalyst

Iron-based catalysts have been gaining popularity in the field of water treatment. In addition to these catalysts being known for their high reactivity with O_3_, abundance in nature, low cost, and toxicity, and the fact that they can be easily synthesized, they also have a large specific surface area, promoting organic contaminants’ adsorption and degradation. Thus, many iron-based catalysts have shown promising results in catalytic ozonation for wastewater treatment [3,14,44,45].

One study investigated the effect of Fe^2+^ and zero-valence iron nanoparticles (nZVI) in catalytic ozonation as a pre-treatment for the biodegradability of complex textile effluents. The kinetic rate constant for the untreated effluent was 0.0152 h^−1^, while for the effluent pretreated with the Fe^2+^ and nZVI catalysts, it was 0.0451 h^−1^ and 0.0569 h^−1^, respectively. This pre-treatment led to an increase in the biodegradability index of up to 0.61 (134.6%) and a higher removal rate of COD (73.5%), color (87%), and toxicity (92%). Pre-treatment with catalyzed O_3_ proved to be a viable option for increasing the biodegradability index, thus promoting the removal of COD, color, and toxicity from the complex textile effluents studied [20].

Ciprofloxacin (CIP) mineralization was studied through heterogeneous catalytic ozonation using ZSM-5 zeolite doped with iron oxide (Fe/ZSM-5 zeolite) as a catalyst. The mineralization rate was improved, with total degradation of CIP after 5 min of the process. The addition of the catalyst resulted in the elimination of 44% of the total organic carbon (TOC) in the first 15 min, in contrast to ozonation alone, where only 13% of TOC removal was achieved in the same period. Fe/ZSM-5 zeolite has been shown to be effective in removing complex non-biodegradable molecules [30].

A nanocatalyst based on α-FeOOH was developed using the non-thermal plasma treatment method to improve the performance of heterogeneous catalytic ozonation in the sulfasalazine antibiotic (SSZ) degradation. After 40 min of treatment, a significant improvement in the removal efficiency (~96.05% DE) of SSZ was achieved when compared to the ozonation process without a catalyst (~61.44% DE). This new nanomaterial demonstrated interesting catalytic properties for efficiently treating wastewater contaminated with SSZ [31,46].

### 2.2. Zinc Oxide

Zinc oxide (ZnO) is widely recognized as an important catalyst in catalytic ozonation. This material has attracted special attention in the field of environmental remediation due to its numerous advantages, such as high catalytic activity, high chemical stability, photosensitivity, excellent adsorption capacity, insolubility in water, and low cost. ZnO can play a crucial role in catalytic ozonation and is one of the most studied catalysts for wastewater treatment [14,47,48].

One study investigated catalytic ozonation using ZnO nanoparticles for the degradation of the antibiotic oxytetracycline (OTC) under the influence of different operating parameters. The results showed that, for the ideal operating conditions (1.38 mg/s at pH = 7), a 94% OTC removal rate was obtained after 35 min of treatment. Ozonation catalyzed by ZnO nanoparticles proved to be a very efficient treatment for the degradation and mineralization of OTC in an aqueous solution [32].

The use of nanometric ZnO was also studied in heterogeneous catalytic ozonation to improve the degradation efficiency of 4-chloro-2-nitrophenol (4C2NP). Using ZnO as a catalyst contributed to an increase in the decomposition rate of 4C2NP, with 98.7% mineralization after 5 min of treatment. It was also observed that the catalytic ozonation process using ZnO at acidic pH conditions (pH = 3) revealed an even higher rate of 4C2NP decomposition compared to alkaline pH conditions. The study showed that the presence of ZnO accelerated the degradation of 4C2NP in the catalytic ozonation process [33].

### 2.3. Aluminium Oxide

As aluminum is one of the most abundant metals in the earth’s crust, aluminum oxide (Al_2_O_3_) can be easily synthesized and used as an efficient catalyst in a wide range of applications, particularly in wastewater treatment. Compared to other metal catalysts, Al_2_O_3_ has a high specific surface area and surface reactivity, promoting the formation of hydroxyl radicals. Catalytic ozonation with aluminum-based materials proves to be an alternative and effective technology for water treatment and has, therefore, been widely applied in various catalytic ozonation systems for the degradation of organic contaminants [29,44,45].

An O@g-C_3_N_4_/Al_2_O_3_ nanocatalyst was developed to improve the efficiency of heterogeneous catalytic ozonation in the treatment of textile wastewater. Adding O@g-C_3_N_4_/Al_2_O_3_ to the catalytic ozonation process resulted in 75% COD removal and 99% color removal after 60 min of treatment. Furthermore, it was found that the BI of the wastewater showed a notable improvement from 0.28 to 0.82 after 90 min of catalytic ozonation. The O@g-C_3_N_4_/Al_2_O_3_ has shown interesting results for full-scale application in the degradation of recalcitrant compounds and emerging contaminants [34].

A magnetic alumina nanocomposite (MANC) was synthesized using a sonochemical method, followed by calcination at 500 °C, to study its catalytic activity in the ozonation process for benzotriazole (BT) degradation. Under optimum conditions, adding 0.5 g of MANC to the ozonation process led to an increase in the rate constant from 0.088 min^−1^ to 0.1957 min^−1^. After adding this catalyst, the time needed to achieve complete COD removal decreased from 25 min to 15 min. Furthermore, MANC showed significant performance in phosphorus removal. This catalyst thus demonstrated significant catalytic activity in the ozonation of BT [35].

Another study investigated the efficiency of the Mn–Ce/Al_2_O_3_ catalyst in the catalytic ozonation system for the degradation of CIP. The results showed that the studied catalyst led to a significant increase in the degradation efficiency of CIP (85.1%) compared to the simple ozonation process (47.4%) after 60 min of treatment. The kinetic constant of CIP degradation was 3 times higher (k = 0.0309 min^−1^) when the catalyst was present in the catalytic ozonation process. Mn–Ce/Al_2_O_3_ has shown considerable potential in developing a highly efficient ozonation process for the degradation of antibiotics present in water [36].

### 2.4. Magnesium Oxide

Magnesium oxide (MgO) has attracted particular attention as a catalyst in catalytic ozonation for the degradation of a wide range of compounds, such as dyes, phenol, 4-chlorophenol, and formaldehyde [44]. MgO stands out for its exceptional properties, such as high surface activity and reactivity, low toxicity, and high structural stability, and it is considered an environmentally friendly material. Thus, MgO has been extensively applied in catalytic ozonation studies for the degradation of different organic compounds in water [14,44,49].

Magnesium oxide (MgO) nanocrystals were used as catalysts in a batch catalytic ozonation reactor to degrade the reactive red 198 azo dye (RR198). The study showed that adding this catalyst to the ozonation process contributed to a significant increase in the degradation rate of RR198, with complete removal of the color after just 9 min, in contrast to the 30 min required for simple ozonation. MgO nanocrystals showed promising results in the catalytic ozonation of dyes, significantly accelerating decolorization and COD removal in the RR198 solution [37].

One group of researchers investigated the efficiency of MgO nanoparticles (MgO-NPs) as a catalyst in an ozonation system for the degradation of 2,4-dichlorophenol (2,4-DCP). The addition of this catalyst to the ozonation process under the optimum conditions (reaction time of 50 min, pH > 7, initial concentration of 2,4-DCP of less than 50 mg/L, and MgO nanoparticle dose of 0.3 mg) led to a removal efficiency of 99.99%. MgO nanoparticles showed high efficiency in the catalytic ozonation of 2,4-DCP [50].

MgO/CNT/graphite nanoparticles were studied as catalysts for wastewater degradation from pesticide factories (PMPW) in a catalytic ozonation process. The results showed that adding the catalyst to the process significantly increased the COD and TOC removal efficiency by 12.73 and 7.11 times compared to those in the sole ozonation. The MgO/CNT/graphite nanoparticles showed excellent catalytic activity in the ozonation processes of organic substances in aqueous solution [38].

### 2.5. Nickel Oxide

Recently, nickel-based catalysts have been studied and applied in catalytic ozonation to degrade various organic pollutants. Nickel oxide (NiO) has some important advantages, namely, high catalytic activity and low cost, compared to noble metals, which makes NiO a suitable option for industrial-scale applications. As such, NiO has proved to be a catalyst with considerable potential in catalytic ozonation, making it possible to achieve higher levels of mineralization of various compounds present in wastewater [51,52].

The catalytic activity of Ni-based layered double hydroxide nanomaterials (Ni-LDHs) in heterogeneous catalytic ozonation for Methyl Orange dye (MO) degradation was studied. The study showed that the addition of the catalyst to the ozonation process, under optimized conditions, achieved degradation kinetics of 0.053 min^−1^ (T = 20 °C) and a COD removal of 72%, in contrast to non-catalytic ozonation (30%), after 60 min of treatment. The Ni-LDH catalyst showed promising results in catalytic ozonation, effectively contributing to an increase in the mineralization of the MO dye [39].

A NiO/MoS_2_ nanocomposite was synthesized using the hydrothermal method and applied to the photocatalytic ozonation process to degrade the antibiotic ciprofloxacin. After adding 0.37 gL^−1^ of NiO/MoS_2_ to the process, total removal of ciprofloxacin (100%) was achieved after 12 min of treatment. The synthesized nanocomposite showed high efficiency as a catalyst in the catalytic ozonation process, and is, therefore, a very interesting approach for the effective degradation of pharmaceutical contaminants present in wastewater [51].

### 2.6. Copper Oxide

The application of copper oxide (CuO) in catalytic ozonation is emerging as an efficient approach to wastewater treatment. CuO is considered a promising catalyst because it contributes to an increase in the oxidation reaction rate and decreases O_3_ consumption, allowing for faster and more complete degradation of organic pollutants and resulting in more effective water treatment. CuO has several advantages, namely, its high chemical stability, low toxicity, and low cost compared to other catalysts. CuO has, therefore, been studied as a catalyst in various catalytic ozonation processes for the degradation of different organic contaminants [3,17,29].

A new copper oxide catalyst supported on carbon aerogel (CuO-Cu_2_O/MCA) was synthesized to evaluate its catalytic properties in the ozonation of simulated textile wastewater for the degradation of the dye Reactive Black 5 (RB-5) in a semi-continuous reactor. The study’s results revealed that, under specific conditions, the percentage of COD removal was 46% in the catalytic ozonation system, in contrast to simple ozonation (29%), after 60 min of treatment. The CuO-Cu_2_O/MCA catalyst showed high efficiency in degrading the RB-5 dye in aqueous solution, indicating that this new catalyst has potential in catalytic ozonation for the treatment of textile wastewater [17].

### 2.7. Silver-Based Catalyst

Silver nanoparticles (AgNPs) have gained recognition as highly active catalysts for degrading organic contaminants in wastewater. Silver (Ag) stands out due to its unique properties, such as its high catalytic activity and powerful antibacterial nature [53,54,55]. Furthermore, AgNPs have high chemical stability, high thermal and electrical conductivity, and a large surface-to-volume ratio [54,56,57]. The combination of these properties makes AgNPs a smart choice for application as nanocatalysts in ozonation for wastewater treatment, promoting the effective removal of organic compounds and contributing significantly to environmental remediation [57,58].

Silver-doped zirconia nanomaterials (Ag/ZrO_2_) were studied as catalysts in ozonation for the degradation of trichlorophenols (TCPs) present in water. The results showed that the addition of the catalyst to ozonation increased the degradation rate of TCPs by 5.32 times (k = 0.0325 min^−1^) compared to the parent ZrO_2_. Ag/ZrO_2_ showed high degradation and reuse capacity in the catalytic ozonation of TCPs [41].

Ag/ZnO nanocomposites were synthesized and applied via photocatalytic ozonation for the degradation and mineralization of phenol. Adding the Ag/ZnO catalyst to the process showed better catalytic performance (k = 0.07 min^−1^) compared to pure ZnO (k = 0.05 min^−1^) in the photocatalytic ozonation of phenol. Ag/ZnO showed promising results in photocatalytic ozonation for water decontamination [42].

Synthesis of bimetallic Ag/Cu nanoparticles supported by MXene (Ag/Cu/MXene) was investigated as an efficient catalyst for the reduction in 4-nitrophenol (4-NP). The Ag/Cu/MXene catalyst exhibited high catalytic activity and good stability, promoting the reduction in 4-NP. Ag/Cu/MXene showed potential for catalytic applications in treating organic pollutants [59].

### 2.8. Gold-Based Catalyst

Among the noble metals, gold nanoparticles (AuNPs) have attracted growing interest from the scientific community in the field of environmental remediation. In recent decades, the application of AuNPs as catalysts in catalytic ozonation has increased given their superior catalytic properties, chemical stability, high selectivity, non-toxicity, and biocompatibility [43,60]. As a result, AuNPs have been widely exploited to promote catalytic reactions and increase efficiency in the degradation and mineralization of a wide range of organic contaminants present in wastewater [60,61,62].

One study investigated the catalytic ozonation of the dye acid Orange 10 (AO-10) in the presence of Au-Bi_2_O_3_ nanoparticles. The addition of Au-Bi_2_O_3_ to the catalytic ozonation process led to a 3.5-fold increase in the degradation rate of AO-10 (k = 7.82 × 10^−3^ s^−1^) when compared to the simple ozonation process (k = 2.29 × 10^−3^ s^−1^). The Au-Bi_2_O_3_ catalyst showed promising results in catalytic ozonation, effectively contributing to increasing the mineralization rate of the AO-10 dye [43].

A Au/TiO_2_ catalyst was synthesized and applied in the photocatalytic ozonation process for the degradation of the pesticide Triclopyr. After adding Au/TiO_2_ to the process, total degradation of Triclopyr (100%) was achieved after 2 h of treatment. Au/TiO_2_ showed high efficiency as a catalyst in the photocatalytic ozonation process, revealing its potential as a promising approach for the effective degradation of pesticides present in wastewater [63].

### 2.9. Manganese Oxide

Manganese oxide-based (MnO) catalysts have been reported as one of the most efficient and economical metal catalysts, with a high capacity to react with O_3_ in the gas phase [44,45]. MnO is a promising material for regulating levels of environmental pollution. Due to its attractive properties, such as high redox potential, ease of synthesis, low cost, low solubility in water, environmental friendliness, abundance in nature, and high specific surface area, MnO has been highlighted in the scientific community as an active heterogeneous catalyst in catalytic ozonation for the degradation of numerous organic compounds present in water [14,29,44].

Catalytic ozonation was studied for deleting metoprolol (MET) and ibuprofen (IBU) using MnO_2_ nanocrystals as a catalyst. To this end, the study evaluated the oxidative reactivity of three different crystalline phases of MnO_2_, corresponding to α-MnO_2_, β-MnO_2_, and γ-MnO_2_. The study’s results revealed that, under specific conditions, the addition of α-MnO_2_ to ozonation led to an increase in the kinetic constant of MET and IBU to values of 0.50 min^−1^ and 0.52 min^−1^, respectively. MnO_2_ nanocrystals have been shown to be effective in catalytic ozonation processes for the degradation of organic pollutants present in wastewater [40].

### 2.10. Titanium Dioxide

The use of titanium dioxide (TiO_2_) as a catalyst is emerging as a promising approach in catalytic ozonation for treating refractory pollutants present in wastewater. TiO_2_ presents high stability in different environments, is of low cost, has high oxidizing power, and has a non-toxic nature [44,64]. Thus, TiO_2_ nanoparticles are suitable for various applications in wastewater treatment. Thus, TiO_2_ is currently one of the most widely used and intensively studied materials in catalytic ozonation [29,44,65].

One study synthesized TiO_2_ nanoparticles using the sol–gel method in order to use them as catalysts in an ozonation system to improve nitrobenzene’s degradation efficiency (NB). The results showed that the addition of the catalyst to the process significantly improved the degradation efficiency of NB when compared to the simple ozonation process. TiO_2_ nanoparticles played a key role in the catalytic ozonation process for the degradation of NB [66].

## 3. Carbon-Based Catalysts

Carbon-based catalysts have been widely researched in catalytic ozonation to treat emerging contaminants. Carbon-based nanomaterials, namely, carbon nanotubes, graphene, and their derivatives, are essentially composed of carbon atoms and stand out for their exceptionally high surface area and selectivity, which makes them the ideal choice for the adsorption and degradation of numerous organic compounds. Therefore, they are very useful in wastewater remediation [3,16,26]. Some examples of carbon-based nanocatalysts used in the ozonation process are summarized in Table 2.

### 3.1. Graphene Oxide

Graphene-based materials have been gaining notoriety in water treatment, starting with the development of effective methods for the degradation of organic pollutants present in water, offering innovative solutions to address significant environmental challenges [22]. Graphene is a substance composed of a single layer of carbon atoms arranged in a hexagonal structure. In addition to its unique structure, graphene’s excellent catalytic capacity is also due to its high specific surface area, high electrical and thermal conductivity, and chemical resistance [29,72].

One of the graphene-based materials that has been gaining popularity is graphene oxide (GO) [29]. Formed by oxidizing graphite, GO can be described as a layer of graphene containing a variety of functional groups where oxygen is bound to its surface. In addition to retaining many of the unique properties of graphene, GO also has other important characteristics, such as high chemical stability and great versatility for a variety of applications in wastewater treatment [29,72]. GO is increasingly being applied for the removal of numerous persistent organic contaminants, including dyes and pharmaceuticals such as antibiotics [72].

In one study, reduced graphene oxides (GOs) doped with N/S and TiO_2_ were synthesized using the Hummers and Brodie methods to test their catalytic properties in the ozonation of oxalic acid. The reduced graphene oxide catalyst doped with nitrogen using the Hummers method was the one that showed the best results in catalyzing the ozonation of oxalic acid (rGOT-HN, k_app_= 9.87 × 10^−3^ min^−1^). The rGOT-HN proved to be a catalyst with potential for the degradation of organic pollutants present in water [67].

### 3.2. Carbon Nanotubes

In the field of nanotechnology, carbon nanotubes (CNTs) have attracted special attention for wastewater treatment due to their excellent chemical, mechanical, and electrical properties. CNTs are carbon allotropes with a cylindrical structure, and depending on the synthesis process, they can be divided into two categories: single-walled CNTs (SWCNTs) and multi-walled CNTs (MWCNTs), which are distinguished by the number of layers (one or many) that make up the nanotubes [29].

CNTs have high chemical and thermal stability, which gives them numerous applications in water treatment [22]. Due to their high specific surface areas, single/multiple layer structure, and hollow nature, CNTs have an excellent adsorption capacity, thus making them promising adsorbents for removing a wide range of organic compounds from water. CNTs have been extensively investigated for the removal of numerous pollutants, including pharmaceuticals, dyes, and pesticides [3,29].

One study investigated the use of manganese oxides supported by carbon nanotubes (MnOx/MWCNT) as catalysts in ozonation for the degradation of CIP in water. The results showed that the synthesized catalyst led to a significant increase in the degradation (87.5% CF degradation in 15 min) and mineralization efficiency of CF. MnOx/MWCNT played a crucial role in heterogeneous catalytic ozonation for the degradation of organic pollutants [68].

Other researchers have used multi-walled carbon nanotubes (MWCNTs) as catalysts in the ozonation of atrazine in aqueous solutions. It was shown that the addition of the catalyst to the catalytic ozonation process led to a decrease in the toxicity of the effluent and an increase in the mineralization of atrazine when compared to the non-catalytic ozonation process. MWCNTs proved to be efficient as catalysts in the ozonation of atrazine in aqueous solution [69].

The use of Fe_3_O_4_/multi-walled carbon nanotubes (Fe_3_O_4_/MWCNTs) as nanocatalysts for the ozonation of p-hydroxybenzoic acid was investigated. The results showed that the addition of the catalyst to ozonation led to a 32% increase in the mineralization rate of p-hydroxybenzoic acid when compared to uncatalyzed ozonation after 5 min of treatment. Fe_3_O_4_/MWCNTs proved to be efficient in the heterogeneous catalytic ozonation of p-hydroxybenzoic acid [70].

### 3.3. Carbon Nanofibers

Carbon nanofibers (CNFs) have also attracted considerable attention in the field of environmental remediation, namely, in the efficient removal of organic contaminants from wastewater. CNFs can be described as cylindrical nanostructures made up of extremely fine carbon fibers and are capable of taking on a variety of shapes. With properties very similar to those of CNTs, CNFs have a high specific surface area, low density, high conductivity, and high porosity. The versatility and exceptional properties of CNFs make them materials of great interest in the field of nanotechnology for water treatment [73,74].

One study developed a catalyst from carbon nanofibers grown on the surface of cordierite monoliths to be applied to the catalytic ozonation of emerging organic micropollutants. The results showed that adding the catalyst to the catalytic ozonation process improved the degree of mineralization of the pollutants studied. The catalyst proved to be an interesting solution for oxidizing emerging organic micropollutants present in water [75].

The catalytic activity of carbon nanofibers doped with nitrogen (N) in ozonation for the degradation of organic pollutants was investigated. This study concluded that the presence of N on the surface of the carbon nanofibers was fundamental to increasing the catalyst’s efficiency in the catalytic ozonation of the organic pollutants studied [76].

Another study developed a catalyst from carbon nanoflakes doped with nitrogen and iron (Fe/D-NC) to be used in the ozonation of *p*-nitrophenol (PNP). The results showed that, under optimal conditions, the addition of the catalyst allowed the degradation efficiency of PNP to reach 90.1% after 30 min of treatment. The PNP degradation rate constant was significantly higher in catalytic ozonation (0.077 min^−1^) than in non-catalytic ozonation (0.012 min^−1^). Fe/D-NC proved to be an efficient catalyst with potential for treating wastewater contaminated with PNP [71].

### 3.4. Carbon Dots

In recent decades, there has been a rapid advancement in nanotechnology, which has stimulated extensive research into the application of carbon-based nanomaterials in catalysis [77]. As a new type of carbon-based nanomaterial, carbon dots (CDs) have gained substantial attention due to their promising catalytic properties and potential applications in various fields of science and technology, especially for environmental purposes [77,78]. CDs have a spherical or almost spherical morphology, with an amorphous or nanocrystalline core and a characteristic size of less than 10 nm [77,79]. CDs stand out for their unique properties, such as good biocompatibility, low toxicity, high photoluminescence, good water solubility, excellent photostability, and chemical stability [79,80]. The synthetic versatility of CDs makes them ideal as catalysts in ozonation for wastewater treatment [77,81,82].

A copper (II)-doped carbon dot (Cu-CD) was developed as a catalyst in a catalytic ozonation advanced oxidation process for the treatment of textile wastewater. To this end, four dyes were analyzed: MO, Orange II sodium salt (O-II), RB-5, and Remazol Brilliant Blue R (RBB-R), as well as a real textile effluent. The presence of Cu-CD increased the apparent degradation rate constants of the dyes—MO (k = 1.184 min^−1^), O-II (k = 1.002 min^−1^), RB-5 (k = 0.709 min^−1^), and RBB-R (k = 0.230 min^−1^)—and of the real textile effluent (k = 0.012 min^−1^) compared to the uncatalyzed reaction. Cu-CD proved to be an efficient catalyst in accelerating ozone decomposition and dye adsorption, showing great potential in textile wastewater treatment applications [15].

## 4. Conclusions

Catalytic ozonation processes offer significant advantages in wastewater treatment, proving to be effective in the degradation of refractory organic compounds. However, there are still some challenges that need to be explored, namely, improving the performance of the processes in terms of degradation efficiency, reducing the costs associated with the treatments, and controlling the formation of secondary by-products so that catalytic ozonation can be applied on a large scale in wastewater treatment.

The use of nanomaterials coupled with ozonation represents a current and promising trend that is gaining momentum globally. Nanocatalysts have high catalytic capacity due to their large specific surface area, abundant active sites, and unique size properties. These unique characteristics allow nanomaterials to be used for a variety of applications in catalysis. Both homogeneous and heterogeneous catalysis have proven to be effective in degrading different compounds. However, using heterogeneous catalysts is preferable since the mineralization of organic pollutants is usually achieved with greater ease of separation and recovery of the catalysts after the process.

Carbon-based nanomaterials are preferred to metal/metal oxide-based nanomaterials in terms of their environmental sustainability, lower levels of toxicity, and efficient use of natural resources, so these nanomaterials will certainly be part of the solution for future water management strategies. There are still some challenges to be faced if nanocatalysts are to reach their full potential in wastewater treatment. Reusing nanocatalysts is a key feature that must be studied in detail, ensuring that the application of nanocatalysts in real conditions is economically viable and environmentally sustainable. Each class of catalysts in catalytic ozonation requires thorough investigation, analysis, and interpretation, given the diversity of catalyst surface performances and the complex intersections between catalysts, O_3_, and organic compounds. Continuous optimization of the synthesis parameters is fundamental in the development of new nanocatalysts to guarantee their viability on an industrial scale. It also needs to be considered that their use in water treatment must not result in damage to the environment. The development of low-cost and high-performance catalysts will continue to be a research priority in the near future.

## Figures and Tables

**Table 1:** Examples of metal/metal oxide-based nanocatalysts used in catalytic ozonation.

Target Pollutants	Catalysts	Rate Constants	Degradation Rate	Reference
Complex textile effluent	Fe^2+^ nZVI	k = 0.0451 h^−1^ k = 0.0569 h^−1^	98.5% decolorization (24 h)	[20]
Ciprofloxacin (CIP)	Fe/ZSM-5 zeolite	k = 0.4129 × 10^−3^ s^−1^	100% CIP degradation (5 min)	[30]
Sulfasalazine (SSZ)	α-FeOOH nanoparticle (PTG-N_2_)	k = 0.076 min^−1^	96.05% SSZ degradation (40 min)	[31]
Oxy-tetracycline (OTC)	Zinc oxide nanoparticles (ZnO)	k = 0.108 min^−1^	94% OTC removal (35 min)	[32]
4-chloro-2-nitrophenol (4C2NP)	Zinc oxide nanoparticles (ZnO)	k = 3.10 × 10^−3^ s^−1^	98.7% 4C2NP degradation (5 min)	[33]
Textile wastewater	O@gC_3_N_4_/Al_2_O_3_	k = 0.1552 min^−1^	99% decolorization (60 min)	[34]
Benzotriazole (BT)	Magnetic alumina nanocomposite (MANC)	k = 0.1957 min^−1^	76% BT removal (6 min)	[35]
Ciprofloxacin (CIP)	Mn–Ce/Al_2_O_3_	k = 0.0309 min^−1^	85.1% CIP degradation (60 min)	[36]
Reactive red 198 azo dye (RR198)	MgO nanocrystal	k = 0.63 min^−1^	100% decolorization (9 min)	[37]
Pesticide manufacturing plant wastewater (PMPW)	nano-MgO/CNT/Graphite	k = 0.021 min^−1^ (COD) k = 0.012 min^−1^ (TOC)	72.41% COD removal (60 min) 51.07% TOC removal (60 min)	[38]
Methyl Orange (MO)	Ni-based layered double hydroxides (Ni-LDHs)	k = 0.053 min^−1^	96% decolorization (60 min)	[39]
Metoprolol (MET) and ibuprofen (IBU)	MnO_2_ nanocrystals (α-MnO_2_)	k = 0.50 min^−1^ (MET) k = 0.52 min^−1^ (IBU)	99.62% MET removal (30 min) 99.51% IBU removal (30 min)	[40]
Trichlorophenols (TCPs)	Ag/ZrO_2_	k = 0.0325 min^−1^	100% TCPs degradation (120 min)	[41]
Phenol	Ag/ZnO	k = 0.07 min^−1^	100% phenol removal (60 min)	[42]
Acid Orange 10 (AO-10)	Au-Bi_2_O_3_	k = 7.82 × 10^−3^ s^−1^		[43]

**Table 2:** Examples of carbon-based nanocatalysts used in the ozonation process.

Target Pollutants	Catalysts	Rate Constants	Degradation Rate	Reference
Oxalic acid	N-doped reduced graphene oxide materials (rGOT-H-N)	k = 9.87 × 10^−3^ min^−1^	96% degradation (15 min)	[67]
Ciprofloxacin (CF)	Carbon nanotube-supported manganese oxides (MnOx/MWCNT)		87.5% CF degradation (15 min)	[68]
Atrazine (ATZ)	Multiwalled carbon nanotubes (MWCNTs)	k = 0.143 min^−1^	75% p-HBA removal (1.5 h)	[69]
p-hydroxybenzoic acid (p-HBA)	Fe_3_O_4_/multi-walled carbon nanotubes (Fe_3_O_4_/MWCNTs)		100% p-HBA removal (10 min)	[70]
p-nitrophenol (PNP)	Metal nitrogen-doped carbon materials (Fe/D-NC)	k = 0.077 min^−1^	90.1% PNP degradation (30 min)	[71]
Methyl Orange (MO) Orange II sodium salt (O-II) Reactive Black 5 (RB-5) Remazol Brilliant Blue R (RBB-R) Real textile effluent	Copper (II) doped carbon dot (Cu-CD)	k = 1.184 min^−1^ (MO) k = 1.002 min^−1^ (O-II) k = 0.709 min^−1^ (RB-5) k = 0.230 min^−1^ (RBB-R) k = 0.012 min^−1^ (effluent)	99.8% MO decolorization (6 min) 99.3% O-II decolorization (6 min) 80.1% RB-5 decolorization (6 min) 99.1% RBB-R decolorization (30 min) 41.0% effluent decolorization (60 min)	[15]
